# Supplementary material for: Spatial validation reveals poor predictive performance of large-scale ecological mapping models
Source: Nat Commun. 2020 Sep 11;11:4540. doi: 10.1038/s41467-020-18321-y (PMC7486894; doi:10.1038/s41467-020-18321-y)
Supplement: Supplementary file 1 — Supplementary Information [file 41467_2020_18321_MOESM1_ESM.pdf]

SUPPLEMENTARY INFORMATION FOR:

Spatial validation reveals poor predictive performance of large-scale ecological mapping models

Ploton, P., et al., Nature Communications, 2020

SUPPLEMENTARY FIGURES

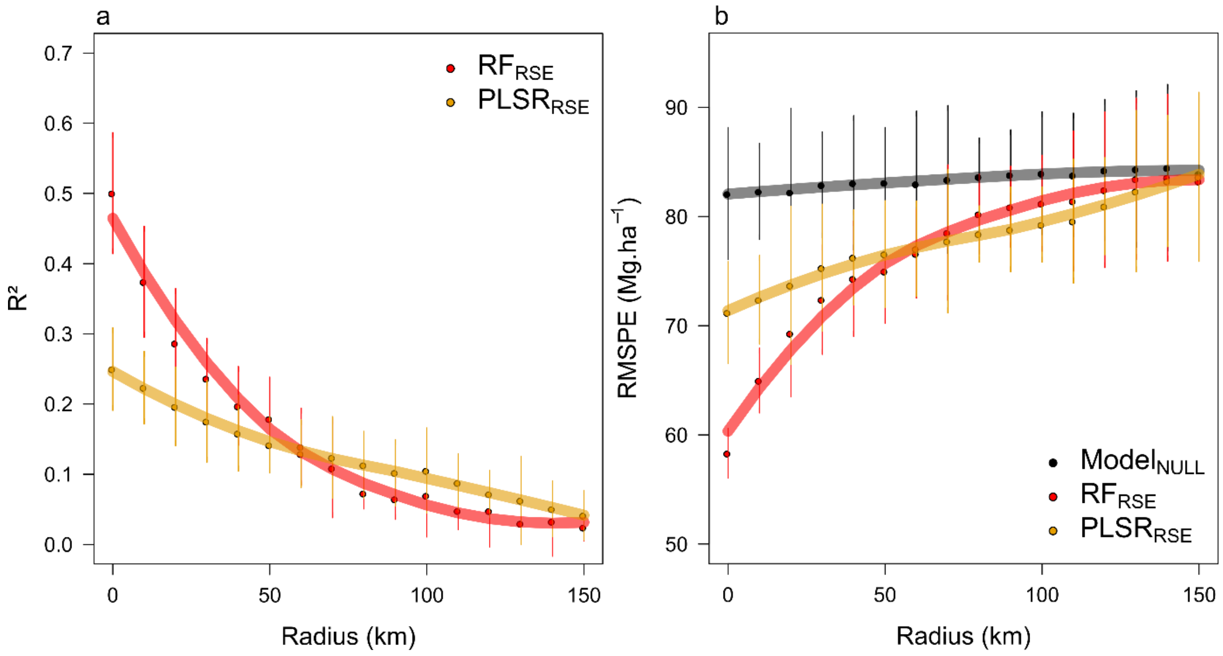

Supplementary Figure 1. Influence of data spatial structure on model's CV statistics. **a**, Change in the coefficient of determination (mean R^2 ± SD over n = 10 iterations, see Methods for details) between predicted and observed pixels' AGB as buffer radius for neighboring pixels exclusion increases in the buffered leave-one-out cross-validation (B-LOO CV). Predictions are made with the Random Forest model based on MODIS and environmental variables (RF_{RSE}, red) and a Partial Least Squares Regression (PLSR) model based on the same set of predictors ($PLSR_{RSE}$, green). **b**, Change in the root mean squared prediction error (mean RMSPE ± SD over n = 10 iterations, see Methods for details) in the B-LOO CV. In addition to RF_{RSE} and $PLSR_{RSE}$, the RMSPE of a null model that systematically predicts the mean of training data is plotted ($Model_{NULL}$, black).

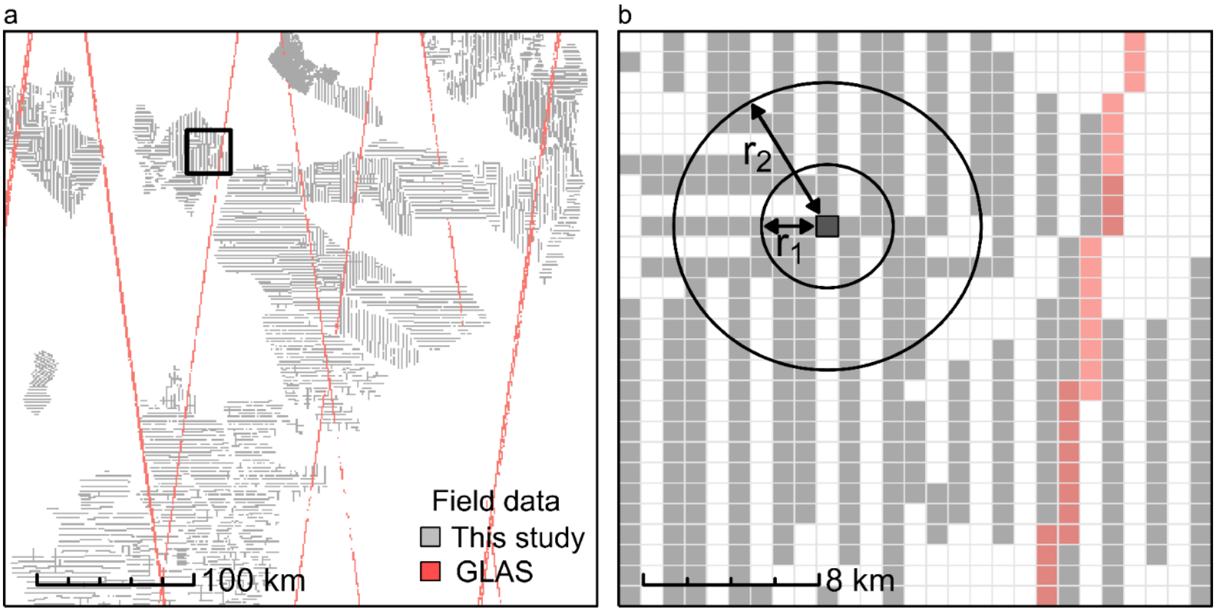

Supplementary Figure 2. Distribution of GLAS data over the illustrative region (**a**) and landscape (**b**) from Figure 3 of the main text. GLAS data were downloaded from the Theia platform (www.theia-land.fr/en/product/lidar/) and correspond to 2003-2005 GLAS shots aggregated to the 1 km resolution.

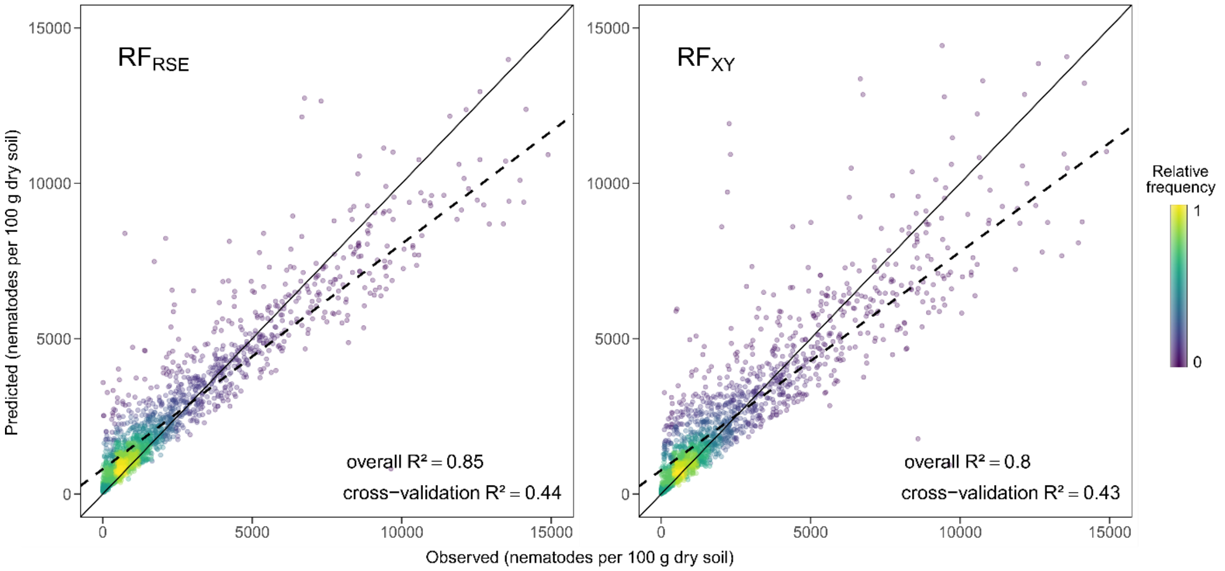

Supplementary Figure 3. Heat plots showing the relationships between predicted versus observed total nematode abundance values for an ensemble model based on 73 covariates (RF_{RSE}, see original publication[1] for details) and a purely spatial ensembled model (RF_{XY}, based on reference data geographic coordinates). Dashed diagonal lines indicate fitted relationships, solid diagonal lines indicate a 1:1 relationship between predicted and observed points. Overall and cross-validation R^2 were computed on the ensemble total nematodes abundance model following the method of the original publication.

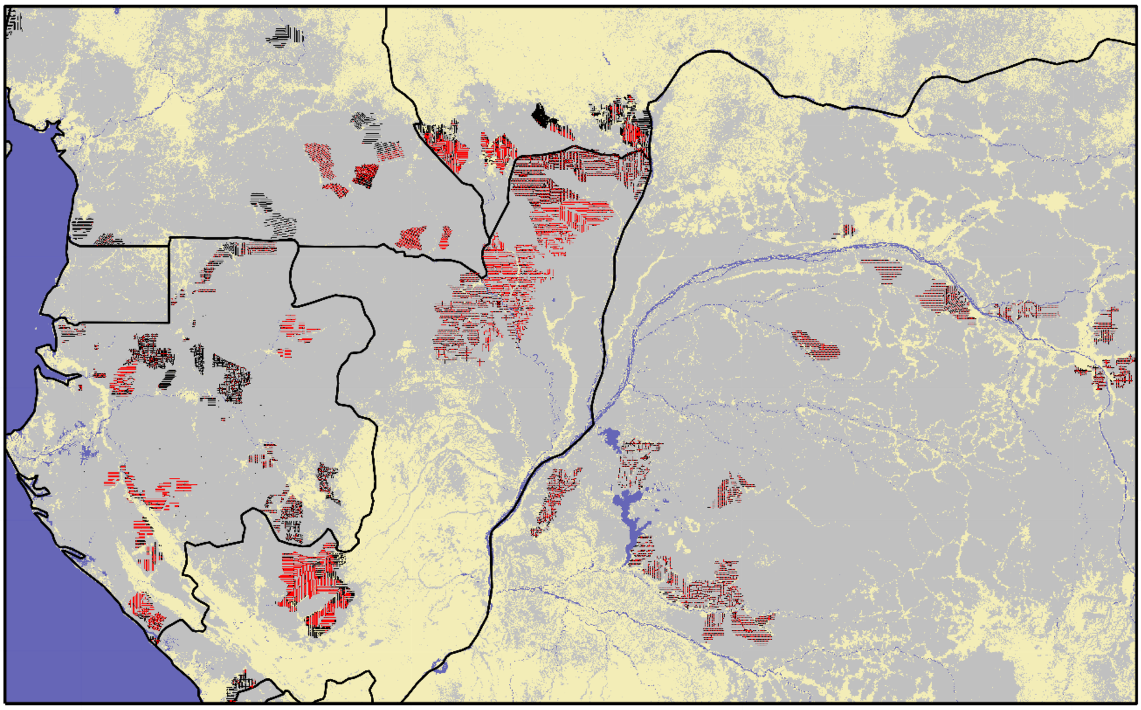

Supplementary Figure 4. Spatial distribution of CoFor-AGB 1 km biomass pixels, with pixels filtered out from the dataset in black and pixels used for biomass modelling in red.

Supplementary References

1. Hoogen, J. van den *et al.* Soil nematode abundance and functional group composition at a global scale. *Nature* **572**, 194–198 (2019).
