# Supplementary material for: Spatial validation reveals poor predictive performance of large-scale ecological mapping models
Source: Nat Commun. 2020 Sep 11;11:4540. doi: 10.1038/s41467-020-18321-y (PMC7486894; doi:10.1038/s41467-020-18321-y)
Supplement: Supplementary file 3 — Reporting Summary [file 41467_2020_18321_MOESM3_ESM.pdf]

nature research

Corresponding author(s): Pierre Ploton

Last updated by author(s): August 11th, 2020

Reporting Summary

Nature Research wishes to improve the reproducibility of the work that we publish. This form provides structure for consistency and transparency in reporting. For further information on Nature Research policies, see our Editorial Policies and the Editorial Policy Checklist.

Statistics

For all statistical analyses, confirm that the following items are present in the figure legend, table legend, main text, or Methods section.

n/a	Confirmed	
[ ]	[x]	The exact sample size (*n*) for each experimental group/condition, given as a discrete number and unit of measurement
[ ]	[x]	A statement on whether measurements were taken from distinct samples or whether the same sample was measured repeatedly
[ ]	[x]	The statistical test(s) used AND whether they are one- or two-sided<br>*Only common tests should be described solely by name; describe more complex techniques in the Methods section.*
[ ]	[x]	A description of all covariates tested
[ ]	[x]	A description of any assumptions or corrections, such as tests of normality and adjustment for multiple comparisons
[ ]	[x]	A full description of the statistical parameters including central tendency (e.g. means) or other basic estimates (e.g. regression coefficient) AND variation (e.g. standard deviation) or associated estimates of uncertainty (e.g. confidence intervals)
[x]	[ ]	For null hypothesis testing, the test statistic (e.g. *F*, *t*, *r*) with confidence intervals, effect sizes, degrees of freedom and *P* value noted<br>*Give P values as exact values whenever suitable.*
[x]	[ ]	For Bayesian analysis, information on the choice of priors and Markov chain Monte Carlo settings
[x]	[ ]	For hierarchical and complex designs, identification of the appropriate level for tests and full reporting of outcomes
[x]	[ ]	Estimates of effect sizes (e.g. Cohen's *d*, Pearson's *r*), indicating how they were calculated

Our web collection on statistics for biologists contains articles on many of the points above.

Software and code

Policy information about availability of computer code

Data collection	Data were organized and managed through the open R statistical software (version 3.5.2).
Data analysis	All data analyses were performed and figures were created with the open R statistical software (version 3.5.2), using the following packages: data.table_1.12.2, rgeos_0.4-2, rgdal_1.4-2, raster_2.8-4, geoR_1.7-5.2.1, caret_6.0-83, randomForest_4.6-14, doParallel_1.0.14, parallel_3.5.2, foreach_1.4.4, sp_1.3-1, ggplot2_3.1.1, ggpubr_0.2, MultivariateRandomForest_1.1.5. Spatial cross-validation technics used in the study have been described in the published literature (e.g. Roberts, D.R. et al., 2017. Ecography ; Valavi, R. et al. 2019. Methods in Ecology and Evolution). The code used for random cross-validation, spatial cross-validation and buffered leave-one-out cross-validation is available through figshare at : https://doi.org/10.6084/m9.figshare.12790085.

For manuscripts utilizing custom algorithms or software that are central to the research but not yet described in published literature, software must be made available to editors and reviewers. We strongly encourage code deposition in a community repository (e.g. GitHub). See the Nature Research guidelines for submitting code & software for further information.

Data

Policy information about availability of data

All manuscripts must include a data availability statement. This statement should provide the following information, where applicable:

- Accession codes, unique identifiers, or web links for publicly available datasets
- A list of figures that have associated raw data
- A description of any restrictions on data availability

All data analyzed in this study are publicly available. The raster of observed AGB pixels is available through figshare (https://doi.org/10.6084/m9.figshare.11865450). Environmental data used in this study were obtained from the following sources: Worldclim (https://worldclim.org/data/worldclim21.html), Global-PET (https:// cgiarcsi.community/data/global-aridity-and-pet-database/), Cloud Cover (http://www.earthenv.org/cloud), SRTM (http://srtm.csi.cgiar.org/srtmdata/) and

Harmonized World Soil Database (HWSD, http://www.fao.org/soils-portal/soil-survey/soil-maps-and-databases). The MODIS MAIAC product cropped over the study area is available through figshare (https://doi.org/10.6084/m9.figshare.12751628). Datasets used to filter pixels were obtained from the following sources: Global Forest Change (https://data.globalforestwatch.org/datasets/tree-cover-2000) and CCI-LC database (Burn Area product v.2.0, 2000-2012 epoch, http://maps.elie.ucl.ac.be/CCI/viewer/download.php). An access link to the nematode worm dataset is provided in the original publication (https://gitlab.ethz.ch/devinrouth/crowther_lab_nematodes). The GLAS data used in Supplementary Figure 2 are available from www.theia-land.fr/en/product/lidar/.

Field-specific reporting

Please select the one below that is the best fit for your research. If you are not sure, read the appropriate sections before making your selection.

☐ Life sciences ☐ Behavioural & social sciences ☒ Ecological, evolutionary & environmental sciences

For a reference copy of the document with all sections, see nature.com/documents/nr-reporting-summary-flat.pdf

Ecological, evolutionary & environmental sciences study design

All studies must disclose on these points even when the disclosure is negative.

Study description	In this study, we use a massive set of dense forest biomass (AGB) estimations derived from management inventories of 113 logging concessions across central Africa. We reproduced a standard approach to map forest AGB, using environmental variables and multispectral images (MODIS) as AGB predictors in a random forest model. We show that the common approach to validate the model (which neglects dependent structures in the data) leads to overly optimistic statistics of model predictive performance. When accounting for dependent structure in the data (i.e. spatial autocorrelation) in model validation (i.e. through a block or “spatial” cross-validation), we found that the model is no more predictive than a simple spatial kriging. This flawed (or insufficient) model validation approach is routinely employed by ‘Big Data’ modelers beyond the realm of AGB mapping. We argue that a spatially explicit assessment of machine-learning model’s predictive power should become the norm in large-scale mapping study.
Research sample	We studied dense tropical forest AGB (i.e. the cumulated biomass of all trees with a diameter at breast height (DBH) >= 10 cm). Our sample is constituted of 59,857 1-km pixels distributed over five countries of central Africa. Pixels AGB estimations were based on a compilation of management forest inventory data, representing c. 200,000 c. 0.5-ha plots (c. 12 million trees measured and identified). A detailed description of pixel AGB computation scheme can be found in the Data descriptor: Ploton, P. et al. A map of African humid tropical forest aboveground biomass derived from management inventories. Sci. Data 7, 221 (2020).
Sampling strategy	At the scale of forest concessions, the sampling strategy of management forest inventories consisted of continuous and parallel transects 20 m or 25 m wide, often 2-3 km apart, and subdivided into rectangular 0.4 or 0.5-ha plots.<br><br>At regional scale, we compiled all available inventories in a single database, namely CoFor. No sample size calculation was performed. CoFor is the largest dataset of forest inventory data ever used to study the spatial distribution of forest biomass in central Africa. It spans from the Atlantic coasts of Gabon and Cameroon to the Democratic Republic of Congo inlands, and thus covers wide regional gradients in terms of forest composition and climate.
Data collection	Data were collected by forest companies in 113 logging concessions in order to build management plans. Most forest companies were assisted and trained by European consultant firms for these inventories (e.g. CIRAD or Foret Resource and Management).
Timing and spatial scale	Data were collected over an area of ca. 160,000 square km from the early 2000s to the early 2010s.
Data exclusions	Our objective was to assess whether commonly used AGB predictors do indeed allow predicting tropical dense forest AGB. We thus filtered a number of pixels to focus the analysis on dense forests (i.e. removing pixels with low forest cover). We also removed a number of pixels where perturbations (i.e. deforestation, fire) may have occurred during the study period (2000-2010), since it may blur the relationship between the level of forest AGB measured in the field and the multispectral covariates (aggregated over 2000-2010). All exclusion criteria were established prior to the analysis. All analyses were made on the filtered dataset.
Reproducibility	All codes from the raw dataset to the results and figures reported in the manuscript were done under the open R statistical software and are available at https://doi.org/10.6084/m9.figshare.12790085
Randomization	Forestry data were acquired through systematic inventories in existing forest concession. No randomization was done.
Blinding	Because data were acquired through a systematic design without any prior stratification or information, data acquisition can be considered here as a blinding process.

Did the study involve field work? ☐ Yes ☒ No

Reporting for specific materials, systems and methods

We require information from authors about some types of materials, experimental systems and methods used in many studies. Here, indicate whether each material, system or method listed is relevant to your study. If you are not sure if a list item applies to your research, read the appropriate section before selecting a response.

Materials & experimental systems

n/a	Involved in the study
[x]	[ ] Antibodies
[x]	[ ] Eukaryotic cell lines
[x]	[ ] Palaeontology and archaeology
[x]	[ ] Animals and other organisms
[x]	[ ] Human research participants
[x]	[ ] Clinical data
[x]	[ ] Dual use research of concern

Methods

n/a	Involved in the study
[x]	[ ] ChIP-seq
[x]	[ ] Flow cytometry
[x]	[ ] MRI-based neuroimaging
